# Healthcare professionals’ perception of parents’ participation in the care of premature infants*

**DOI:** 10.1590/1980-220X-REEUSP-2025-0532en

**Published:** 2026-06-26

**Authors:** Adrielly Cristina de Lima Raimundo, Ana Mirelle dos Santos, Paulyne Souza Silva Guimarães, Ingrid Martins Leite Lúcio, Ana Virgínia Rodrigues Veríssimo, Ana Carolina Santana Vieira, Rossana Teotônio de Farias Moreira

**Affiliations:** 1 Universidade Federal de Alagoas, Escola de Enfermagem, Programa de Pós-graduação em Enfermagem, Maceió, AL, Brazil.; 2 Universidade Federal de Alagoas, Escola de Enfermagem, Maceió, AL, Brazil.; 3 Universidade de Pernambuco, Faculdade de Enfermagem Nossa Senhora das Graças, Recife, PE, Brazil.

**Keywords:** Infant, Premature, Intensive Care Units, Neonatal, Family, Health Personnel

## Abstract

**Objective:**

To describe parents’ participation in the care of premature newborns from the perspective of professionals working in the Neonatal Intensive Care Unit.

**Method:**

A descriptive qualitative study conducted in March 2025 with 26 professionals from the multidisciplinary team working in two Neonatal Intensive Care Units located in Maceió, Alagoas. The study was submitted to the Research Ethics Committee of the Universidade Federal de Alagoas, under Opinion 7,182,417.

**Results:**

From the analysis of the interviews, two categories emerged: “Between fear and discovery” and “Providing care and promoting bonding”. Professionals reported the inclusion of parents in the unit’s routine to reduce fear and expand benefits for the infant, especially through the kangaroo method.

**Conclusion:**

Fear and uncertainty hinder parents’ inclusion in care. Welcoming, guidance, and encouragement of care practices by the healthcare team may help overcome these barriers. Humanization policies are necessary to ensure shared responsibility in care.

## INTRODUCTION

Premature birth is an event that imposes significant challenges on families and healthcare professionals, especially when a newborn (NB) requires intensive care in Neonatal Intensive Care Units (NICUs). Given the complexity of this scenario, there has been increasing appreciation of practices that promote parents’ active involvement in the care of their premature infant, recognizing them as a fundamental part of a child’s recovery and developmental process^(1)^.

The hospitalization of an infant in the NICU is a stressful experience for parents, which may generate emotions related to fear and loneliness due to distancing and lack of preparedness. However, when support from a multidisciplinary team is present, these feelings may be replaced by self-confidence^(2)^.

In this context, the role of the healthcare team is to act as a mediator between the technical environment and the emotional dimension experienced by parents. The way professionals welcome, guide, and integrate the family into care directly impacts the quality of parents’ experience during their child’s hospitalization. This premise constitutes the core of Family-Centered Care, a care model that recognizes the family as an essential partner in the NB’s care and recovery process^(3)^. Nevertheless, despite advances in humanization policies, institutional, cultural, and communication barriers still persist, limiting parents’ effective participation in neonatal care^(4)^.

Neonatal care practices that encourage family inclusion and closeness in the NB’s daily routine during hospitalization have been promoted with the aim of strengthening bonding and child development. In Brazil, the kangaroo method stands out as one of the humanized care strategies for critically ill or potentially critically ill NBs, encompassing biopsychosocial intervention plans focused on the infant and their family^(5)^.

However, studies still demonstrate that mothers’ lack of preparedness to care for NBs who remained hospitalized for long periods in neonatal units may negatively impact post-discharge childcare. Many parents report feelings of hesitation, fear, and unpreparedness regarding routine childcare activities, such as diaper changing. This demonstrates that the guidance provided by healthcare professionals directly influences the infant’s health within home settings^(6)^.

In care practice, it is observed that the multidisciplinary team in a NICU, including nurses, requires technical-scientific knowledge and training to enhance the quality of care provided to NBs and families. During the care process, it is essential that professionals understand the mother–infant relationship as positive for preserving and restoring the NB’s health, as well as recognizing parents’ needs in order to provide individualized care^(7)^.

Thus, the present study seeks to answer the following question: how do you, as a professional working in a NICU, perceive family participation in the care of premature NBs, considering the seven standard neuroprotective measures? This study is relevant not only for improving care practices, but also for supporting public policies aimed at humanizing care and promoting the comprehensive health of the child and their family, contributing to the construction of a care model centered on family and shared responsibility. Therefore, the study aims to describe parents’ participation in the care of premature NBs from the perspective of professionals working in the NICU.

## METHOD

### Study Design

This is a descriptive study with a qualitative perspective conducted with the multidisciplinary team working in NICUs, based on Altimier and Phillips’ Developmental Care Model^(8)^. This model was applied in the construction of the interview script for data collection, as well as in data analysis and discussion, focusing on the axis of parent participation. The model presents a framework that guides clinical practice in NICUs toward neuroprotection through seven developmental care measures represented by a lotus flower. These measures involve the healing setting, optimization of nutrition, minimization of pain and stress, skin protection, safeguarding sleep, positioning and handling, and partnership with families, with the mother–infant dyad positioned at the center of the flower. The study followed the recommendations of the COnsolidated criteria for REporting Qualitative research.

### Setting, Population, and Selection Criteria

This study was conducted in two NICUs from two teaching hospitals, which together total 36 beds and provide care through the Unified Health System, both located in Maceió, Alagoas, Brazil. Professionals from the multidisciplinary team who provided direct care to NBs were included, such as physicians, nurses, physiotherapists, speech therapists, and nursing technicians, provided they had at least one year of professional experience in the unit.

### Data Collection

Data collection was conducted in March 2025 through individual interviews with healthcare professionals. A convenience sampling approach was adopted, respecting participants’ availability during the data collection period: the researcher attended the units twice a week and, according to the active duty schedule, presented the study proposal and invited professionals to participate voluntarily. All interviews were conducted individually in a private setting, ensuring participant confidentiality and anonymity.

Considering the routine of the units and the need to reduce potential discomfort, the interviews were conducted in two formats: written, through delivery of the form for later completion and return by the professional; or recorded, with audio capture during application of the previously presented, explained, and authorized interview script. Both interview formats followed the same question guide. The following triggering question was used: how do you, as a professional working in a NICU, perceive family participation in the care of premature NBs, considering the seven standard neuroprotective measures?

### Data Analysis and Processing

Data obtained from written forms and recorded interviews were organized into standardized digital documents. Textual responses and audio recordings were transcribed literally and in full, preserving the original content. Subsequently, the information was organized into tables and analyzed through Bardin’s content analysis^(9)^. Analysis of the interviews demonstrated that there was no loss of content regardless of the data collection format used.

### Ethical Aspects

The study was approved by the Research Ethics Committee of *Universidade Federal de Alagoas*, under Opinion 7,182,417. All professionals signed the Informed Consent Form, ensuring anonymity and voluntary participation. To identify participants while preserving anonymity, the letter “P” followed by Arabic numerals in ascending order was used (P1, P2, P3, and so forth).

## RESULTS

A total of 26 professionals from the multidisciplinary team participated in this study, from a universe of 300 professionals, including physicians, nurses, physiotherapists, speech therapists, and nursing technicians. The main topics addressed involved the impact of NICU hospitalization, participation in care, parents’ feelings, bonding, and welcoming within the NICU, which, in the Developmental Care Model, may be related to one of the lotus petals concerning partnership with the family and to the center representing the mother–infant dyad. The results were organized and discussed according to two categories: “Between fear and discovery” and “Providing care and promoting bonding”.

### Between Fear and Discovery

The NICU hospitalization period is challenging for both parents and infants from its very beginning. Thus, parents’ first contact with their infant often occurs in the ICU, an unfamiliar and frightening setting in which the professional acts as a mediator of this encounter:

*Welcoming and offering the initial contact* (P4); *Because I think the ICU setting, simply because it is an ICU, is a name that scares people and makes them afraid. So, each person has their own time to create a bond with the child, because there is still that issue of the ideal baby and the real baby* (…) *she arrives in the ICU and still needs to establish bonding, which contributes to development* (P21).

From that moment on, as parents gradually become familiar with the daily routine and processes, they slowly adapt to the setting, which is still new, to the routine, which is still undefined, and to the NB’s clinical condition, which is complex. Therefore, fear permeates their stay beside the infant. Many interviewed professionals mentioned the “fear” parents experience, as observed in the following statements:

*They are afraid to touch* (P3). *The family is very afraid. Fear of loss, fear of worsening, fear of severity, even fear of touching the NB* (P24); *The family usually just observes* (P23); (…) *the first impact is something we know is very intense, right? Then they gradually adapt and get used to it* (P22).

Within this context of overcoming the initial contact and the maternal condition, which still requires attention, the mother becomes involved in caring for her infant and is incorporated into a new setting, adapting to feeding schedules and care routines for her infant. Little by little, they begin to engage and get closer to the NB:

*They are afraid, we ask whether they already know how to change diapers, but they are afraid to handle the baby because most of them are monitored, so they do not know how to handle the infant* (P17); (…) *mothers are afraid to touch the baby… we talk to them, encourage touch, encourage contact with the infant* (…) *it is a challenge* (P20).

Throughout the entire hospitalization process, regardless of the length of NICU stay, the importance of information and the benefits of parents remaining beside the infant are emphasized, considering that this hospitalization period also occurs during a moment of maternal sensitivity and fragility. Often, this period becomes a milestone for the establishment of bonding, and the more information parents have available and the more informed they are about their child’s needs and care, the smoother this experience becomes:

*Providing information about the disease and answering questions* (P6); *They are extremely anxious people, in need of information and attention. They become calmer and participate more in the healing processes whenever they are better informed and welcomed* (P7); *Most family members remain present, seek information, and are interested in practicing the kangaroo position* (P9); (…) *I notice it more regarding breastfeeding because the mother becomes more bonded to her infant, but the father is more the one who only looks and observes* (P12).

Thus, as this interaction develops, family members and the healthcare team come together in favor of the NB’s care, strengthening trust, participation, and engagement in the infant’s recovery:

*There is a trajectory of learning and immersion together with the team in caring for the infant — a very individual trajectory, in which the family is included in the care and has unrestricted access to their children* (P8); *Most participate interactively in the NB’s care, offering feeding, kangaroo positioning, and receiving information about the clinical condition* (P10).

Another aspect described by participants concerned bonding, considering the distance generated by the infant’s instability, the quantity of devices, the incubator itself, and the need for intensive care. Mothers become distant from the care they would normally provide to their infants during this period and, consequently, have difficulty perceiving themselves as mothers in those moments:

*They often say that sometimes they feel the child does not belong to them. When we place the infant in the kangaroo position, I have often heard, “This is the first time I feel like I am being my child’s mother, that my child belongs to me”* (P11); (…) *they become afraid when they see the baby inside the incubator. But it is the same baby they would have at home with them; the difference is that here the baby needs our care, but the baby still belongs to them* (P25).

The construction of bonding, which is so important for child growth and development, ends up occurring relatively late and progresses more slowly due to the need to overcome fears, doubts, and anguish:

*The kangaroo position, both for the father and the mother* (…) *the interaction of touching the infant and talking to them. Because they are premature NBs, sometimes parents become afraid, but then I encourage them* (…) *because it is good for the NBs, right? For their development* (P18).

One of the first barriers to parents’ participation in care and bonding with the NB in the ICU is the infant’s clinical condition, considering their instability and need for intensive care, as illustrated in the following statement:

*At first, they are afraid. Because either the babies are on ventilators or there is some complication — many times the baby experiences desaturation, and then they end up associating the infant’s instability with kangaroo care, when actually it is because the infant is more critically ill* (P1).

Other professionals reinforced what had previously been reported:

*And then they gradually adapt to that care, and they end up losing their fear, getting used to the noise and the drops in oxygen saturation when they begin to understand the situation* (P2).

Thus, according to the professionals’ statements, it can be observed that the initial shock caused by the NICU setting becomes less impactful over the hospitalization period. Parents remain vigilant regarding the infant’s symptoms, but partnership with the healthcare team allows this experience to become smoother, as they are gradually included in care and, even more importantly, informed throughout the hospitalization process.

### Providing Care and Promoting Bonding

Due to their daily routines, mothers tend to remain more present at the infant’s bedside because of the milk expression process to feed the infant and the possibility of remaining hospitalized, considering the benefits of their presence for the NB:

*Our closest interaction is with the mothers, who remain as companions in the hospital and routinely come for human milk expression and kangaroo positioning. And the team has increasingly recognized the importance of this. But I think there is still much progress to be made regarding participation in care* (P14); *The family is included in treatment — immunotherapy and skin-to-skin contact* (P5).

Participation in care is relative and varies from one mother to another. However, it is worth emphasizing that as the team reinforces the importance of participating not only in care and milk expression, but also simply in being present in the unit, this already makes a difference for the infant’s health:

*I realize that if we explain the importance of their greater participation in the infant’s care, they recognize the importance of their presence and become more interested in participating in care together with us* (P19).

Opportunities for participation in the infant’s care may vary, ranging from touch and kangaroo positioning to diaper changing. These care practices include both those specific to a neonatal unit, such as feeding through a tube, and those closer to family care, such as diaper changing:

*Sometimes it happens that while we are administering the feeding, we ask them to hold the syringe at that moment during feeding. But this is rare in the ICU. Their participation is really more therapeutic touch and kangaroo positioning* (P2).

During these moments of participation, new possibilities also emerge, considering the role professionals play in relation to the guidance they can provide to the family in this particular type of care:

*I ask the mother to help change a diaper, and then I teach her how to change it. I try as much as possible to encourage kangaroo positioning, to talk with her, and to help her understand that the child belongs to her* (P11); *They need guidance regarding strategies to promote neuroprotection* (P26).

In addition to the father and mother, who have unrestricted access to the unit and to their infants, other family members have limited access to the units for visiting the infant, but they do not participate much in the infant’s care:

*The family does not have access to the unit, only grandparents or another family-linked guardian, once a week, which prevents/makes difficult the development of bonding and care with the child* (P15); (…) *it is rare for me to see families becoming deeply involved… there are very few cases* (P13).

Professionals’ perceptions regarding participation in care are diverse and vary according to parents’ availability, fears, and the infant’s clinical stability. Their statements mainly highlight participation in kangaroo positioning and milk expression for feeding administration. Grandparents may visit the infants, but they do not participate in their care.

For some professionals, visualizing this participation in care is still difficult, and they perceive that such participation would be more tangible in other units through which the infant passes, considering the continuity of care and the infant’s instability:

*In the ICU, I still think it is very limited* (…) *I think they only begin participating more in care in the kangaroo unit. But that is not ideal, because we should promote this participation from the ICU onward* (P11).

Another challenge concerns family guidance, as stated below:

*Most of the time it is the mother who becomes more involved in everything related to the infant* (…) *and there are mothers who are more distant, sometimes even due to lack of information and guidance, which sometimes they do not receive. Sometimes this information is lacking* (…) *rights they have but are not informed about* (P14).

However, this is often related to the educational level of that woman/family:

*We receive mothers/families from different contexts… from planned pregnancies to unwanted pregnancies* (…) *with higher and lower educational and income levels* (P16).

Professionals pointed out the need for a more attentive perspective toward the mothers of these infants, who become accompanying mothers during hospitalization in the unit and follow a demanding routine of bedside manual milk expression every three hours, which may be emotionally and physically exhausting. Some professionals still perceive more restricted participation in care, whereas others report greater interaction and exchange between the mother and her infant during care. Finally, emphasis is placed on the role of guidance for these mothers/fathers within the care context, highlighting the importance of adapting communication to their understanding.

## DISCUSSION

Premature birth causes motherhood itself to occur prematurely, leaving women without time to assimilate and adjust the representations of the baby who arrived, making adaptation to this new reality necessary. After the unplanned separation, the mother encounters in the NICU a fragile, thin infant surrounded by equipment, differing greatly from what she had imagined^(10)^.

NICU hospitalization is challenging for parents because it compromises family dynamics and distances parents from the NB’s primary care, generating insecurity and helplessness. Developing parenthood surrounded by noise, constant lighting, and unfamiliar people outside one’s social circle may transform this experience^(11)^, and therefore it may be permeated by feelings such as frustration, guilt, and maternal distancing^(12)^. This corroborates what professionals in this study reported regarding families’ fear of NICU hospitalization.

The infant’s need for hospitalization causes a shift in the role of primary caregiver, which becomes assumed by healthcare professionals, potentially generating feelings of stress, anxiety, and depression in parents^(13)^. Consequently, this process hinders bonding between parents and infants, as mentioned by P11 when reporting that mothers feel closer to their infants when placing them in the kangaroo position. The presence of family members in the NICU promotes interaction between mother and infant, which positively impacts the preterm newborn’s (PTNB) brain. Skin-to-skin contact is considered the best environment for the NB because it creates a sensory environment with beneficial interactions for both, helping to minimize pain, promote bonding, and facilitate thermoregulation^(8)^.

The absence of information regarding the infant’s clinical condition may also distance mothers from their child, increasing parental stress and anxiety levels. Therefore, it is essential that professionals in the unit act as support, helping parents observe and understand the signals their child expresses during moments of interaction^(14)^.

Over time, through interaction between mother and team and through adaptation to the unit routine, changes in maternal behavior become perceptible, which may be associated with overcoming initial feelings, welcoming by the team, and the sense of security that helps strengthen the bond among professionals, family members, and children^(12)^. However, the infant’s clinical instability, combined with the possibility that the infant may not survive hospitalization, still generates feelings of fear, insecurity, and anguish. These feelings may intensify depending on the child’s health condition^(10)^.

It is worth emphasizing that public policies guarantee parents’ participation in childcare during hospitalization, beginning in the periods of pregnancy, childbirth, and postpartum, including partner prenatal care and the right to a companion of free choice, as well as unrestricted access and permanence with the NB in the neonatal unit^(15)^. Equally important, the information received regarding the infant’s health condition may help parents understand the hospitalization process and later assume home care more safely^(14)^.

Parents’ participation in care is capable of reducing the impact of NICU hospitalization on the NB’s development and constitutes one of the pillars of developmental care. Skin-to-skin contact is a measure that helps relieve pain, provides a sense of security for the dyad, facilitates breastfeeding, and reduces stress. In contrast, separation from parents may negatively affect PTNB development and the establishment of bonding^(16)^.

Ensuring parents’ participation in care together with the PTNB is another relevant factor associated with better outcomes. In units with parental care programs, reductions in family stress, increased breastfeeding rates, and improved neurological development and weight gain outcomes for the infant have been observed. Through active participation in care, parents also demonstrated acquisition of knowledge regarding hygiene and increased maternal self-confidence in caring for their child^(17)^.

Furthermore, access to information regarding the NB’s hospitalization may reduce negative feelings experienced by parents during this period, helping them feel involved and prepared to care for their child^(18)^. Authors who assessed parental stress among parents of infants hospitalized in NICUs demonstrated that, when informed about the care their children were receiving, parents experienced lower stress levels associated with hospitalization, reinforcing that during this period parents require support, assistance, and guidance from the healthcare team^(19)^.

Moreover, researchers have also emphasized the importance of supporting mothers regarding how to interact with their infant, and not only regarding the infant’s clinical condition. Such information may help reduce maternal insecurity and increase positive perceptions and well-being. Another beneficial factor is the kangaroo method, which strengthens awareness of the maternal role and stimulates early communication, contributing to more positive perceptions of the hospitalization period^(20)^.

The main forms of mothers’ participation in the care of premature infants include physical presence, monitoring the care performed by the healthcare team, seeking information about the infant, hygiene and feeding care, touch, holding the infant, and kangaroo positioning^(21)^. The professionals interviewed in this study stated that participation occurs mainly in relation to kangaroo positioning, feeding administration, and presence in the unit.

Including parents in the care of premature infants in NICUs remains a challenge, as described by many authors^(5,12,15)^. However, it is the responsibility of the unit’s multidisciplinary team to act as an intermediary in establishing bonding and closeness between parents and infant, contributing to the construction of care that will facilitate a smoother transition to discharge for both the healthcare team and the family^(22)^. One of the axes of developmental care is precisely the role of the healthcare team in empowering and promoting this participation, contributing to practices within the model that have the potential to minimize the consequences of NICU hospitalization^(17)^.

## CONCLUSION

From professionals’ perspective, parents’ participation in the care of premature NBs in the NICU is a complex and dynamic process. Initially, fear and uncertainty hinder bonding and inclusion in care. These barriers are overcome through the work of the multidisciplinary team by means of welcoming, continuous guidance, and encouragement of practices such as the kangaroo method, which transforms fear into confidence.

At the core of the Developmental Care Model, despite being directly related to one of the seven presented axes, family participation is transversal to the other “petals” of the “lotus flower” representing the model, enriching the PTNB’s sensory and interactional environment, reducing pain and stress through comfort, breastfeeding, and reduction of pain responses, while promoting thermoregulation, skin-to-skin contact, and encouragement of breastfeeding.

However, challenges persist, such as the NB’s clinical instability, limited participation of family members, and socioeconomic and institutional influences. Thus, there is a need for humanization policies prioritizing qualified communication and emotional support, including parents as co-responsible participants from admission onward. This approach is essential for building stable family bonds and ensuring sustainable care after hospital discharge.

## Data Availability

The entire dataset supporting the results of this study is part of a dissertation research project that has not yet been fully published.
