# Supplementary material for: Healthcare professionals’ perception of parents’ participation in the care of premature infants
Source: Rev Esc Enferm USP. 2026 Jun 26;60:e20250532. doi: 10.1590/1980-220X-REEUSP-2025-0532en (PMC13313085; doi:10.1590/1980-220X-REEUSP-2025-0532en)
Supplement: Translation

# Translation: Percepção dos profissionais de unidade neonatal sobre a participação dos pais no cuidado ao prematuro*

**DOI:** 10.1590/1980-220X-REEUSP-2025-0532pt

**Authors:** Adrielly Cristina de Lima Raimundo, Ana Mirelle dos Santos, Paulyne Souza Silva Guimarães, Ingrid Martins Leite Lúcio, Ana Virgínia Rodrigues Veríssimo, Ana Carolina Santana Vieira, Rossana Teotônio de Farias Moreira

**Affiliations:** 1 Universidade Federal de Alagoas, Escola de Enfermagem, Programa de Pós-graduação em Enfermagem, Maceió, AL, Brasil.; 2 Universidade Federal de Alagoas, Escola de Enfermagem, Maceió, AL, Brasil.; 3 Universidade de Pernambuco, Faculdade de Enfermagem Nossa Senhora das Graças, Recife, PE, Brasil.

**Keywords:** Recém-Nascido Prematuro, Unidades de Terapia Intensiva Neonatal, Família, Pessoal de Saúde

## Abstract

**Objetivo:**

Descrever a participação dos pais no cuidado ao recém-nascido prematuro na perspectiva dos profissionais da Unidade de Terapia Intensiva Neonatal.

**Método:**

Estudo descritivo e qualitativo, realizado em março de 2025 com 26 profissionais da equipe multiprofissional atuantes em duas Unidades de Terapia Intensiva Neonatal localizadas em Maceió, Alagoas. O estudo foi submetido ao Comitê de Ética em Pesquisa da Universidade Federal de Alagoas, sob Parecer n° 7.182.417.

**Resultados:**

A partir da leitura das entrevistas, emergiram duas categorias: “Entre o medo e a descoberta” e “Executando cuidados e promovendo o vínculo”. Os profissionais relataram a inserção dos pais na rotina da unidade para reduzir o medo e ampliar os benefícios ao bebê, principalmente com o método canguru.

**Conclusão:**

O medo e a incerteza dificultam a inserção dos pais nos cuidados. O acolhimento, a orientação e o incentivo às práticas pela equipe podem ajudar a superar. Políticas de humanização são necessárias para assegurar a corresponsabilidade no cuidado.

## INTRODUÇÃO

O nascimento prematuro é um evento que impõe desafios significativos à família e aos profissionais de saúde, especialmente quando o recém-nascido (RN) necessita de cuidados intensivos em Unidades de Terapia Intensiva Neonatal (UTINs). Diante da complexidade desse cenário, cresce a valorização de práticas que promovam o envolvimento ativo dos pais no cuidado ao filho prematuro, reconhecendo-os como parte fundamental no processo de recuperação e desenvolvimento da criança^(1)^.

O processo de hospitalização de um bebê na UTIN é uma vivência estressante para os pais, o que pode ocasionar emoções relacionadas ao medo e à solidão diante do distanciamento e despreparo. No entanto, quando existe o suporte de uma equipe multiprofissional, esse sentimento pode ser substituído pela autoconfiança^(2)^.

Nesse contexto, o papel da equipe é atuar como mediadora entre o ambiente técnico e a dimensão emocional dos pais. A forma como os profissionais acolhem, orientam e integram a família ao cuidado impacta diretamente a qualidade da experiência vivida pelos pais durante a internação do filho. Essa premissa constitui o cerne do Cuidado Centrado na Família, modelo assistencial que reconhece a família como parceira essencial no processo de cuidado e recuperação do RN^(3)^. No entanto, apesar dos avanços nas políticas de humanização, ainda persistem barreiras institucionais, culturais e comunicacionais que limitam a efetiva participação dos pais no cuidado neonatal^(4)^.

As práticas de assistência neonatal que trabalham com a inclusão e aproximação da família no dia a dia do RN durante a etapa de internação em unidade hospitalar têm sido propagadas com o intuito de fortalecer a afetividade e o desenvolvimento infantil. No Brasil, tem-se o método canguru como uma das estratégias de cuidado humanizado aos RNs graves ou potencialmente graves, englobando planos de intervenções biopsicossociais focados na atenção ao bebê e sua família^(5)^.

Entretanto, estudo ainda demonstra que o despreparo das mães em cuidar do RN que permaneceu por longo tempo internado em unidades neonatais pode impactar o cuidado pós-alta da criança. Diversos pais afirmam sensações de hesitação, temor e despreparo diante de ações de rotina relacionadas ao filho, como troca de fraldas. Isso demonstra que as orientações passadas por profissionais repercutem diretamente na saúde do bebê no ambiente doméstico^(6)^.

Na rotina assistencial, observa-se que a equipe multiprofissional de uma UTIN, incluindo o enfermeiro, precisa de fundamentos técnicos científicos e de capacitação para potencializar a qualidade dos cuidados aos RNs e familiares. No processo do cuidado, é fundamental que o profissional compreenda a relação mãe-bebê como positiva para a preservação e reabilitação da saúde do RN, assim como as necessidades dos pais, com o intuito de oportunizar uma assistência individualizada^(7)^.

Assim, o presente estudo busca responder ao seguinte questionamento: como você, enquanto profissional atuante em UTIN, observa a participação dos familiares no cuidado ao RN prematuro, considerando as sete medidas padrão neuroprotetoras?”. Torna-se relevante não apenas para qualificar as práticas assistenciais, mas também para subsidiar políticas públicas voltadas à humanização do cuidado e à promoção da saúde integral da criança e de sua família, com vistas a contribuir para a construção de um modelo de cuidado centrado na família e na corresponsabilidade. Propõe-se, portanto, descrever a participação dos pais no cuidado aos RNs prematuros na perspectiva dos profissionais na UTIN.

## MÉTODO

### Tipo de Estudo

Trata-se de estudo descritivo, de perspectiva qualitativa, realizado com a equipe multiprofissional atuante em UTIN à luz do Modelo de Cuidado Desenvolvimental de Altimier e Phillips^(8)^. Esse modelo foi aplicado na construção do roteiro de entrevistas para coleta de dados, na análise e na discussão dos dados, focando no eixo de participação dos pais. O modelo apresenta uma estrutura que orienta a prática clínica em UTIN para a neuroproteção, a partir de sete medidas para o cuidado desenvolvimental, representadas por uma flor de lótus. Essas medidas envolvem o ambiente de cura, a otimização da nutrição, a minimização da dor e do estresse, a proteção da pele, a salvaguarda do sono, o posicionamento e o manuseio, e a parceria com as famílias, sendo a díade mãe-bebê o centro dessa flor. O estudo seguiu as recomendações do *COnsolidated criteria for REporting Qualitative research*.

### Local, População e Critérios de Seleção

Este estudo foi realizado em duas UTINs de dois hospitais-escola, que juntas totalizam 36 leitos, com atendimento pelo Sistema Único de Saúde, ambos localizados em Maceió, Alagoas, Brasil. Foram incluídos no estudo os profissionais da equipe multiprofissional que prestam assistência direta no cuidado ao RN, como: médicos, enfermeiros, fisioterapeutas, fonoaudiólogos e técnicos de enfermagem; e que atuassem no mínimo por um ano.

### Coleta de Dados

A coleta de dados foi realizada em março de 2025, por meio de entrevistas individuais com profissionais de saúde. Optou-se por uma abordagem de amostragem por conveniência, respeitando a disponibilidade dos participantes no período de coleta: a pesquisadora comparecia às unidades duas vezes por semana, e conforme a escala de plantão vigente, apresentava a proposta do estudo e convidava os profissionais a participarem voluntariamente. Todas as entrevistas ocorreram de forma individual, em local reservado, garantindo o sigilo e o anonimato dos participantes.

Considerando a rotina das unidades e a necessidade de reduzir possíveis constrangimentos, as entrevistas foram conduzidas de duas formas: escrita, com entrega do formulário para posterior devolução preenchido pelo profissional; ou gravada, com captura de áudio durante a aplicação do roteiro, previamente apresentado, informado e autorizado pelo profissional. Ambas as formas de entrevista seguiram o mesmo roteiro de perguntas. Utilizou-se como questão disparadora: como você, enquanto profissional atuante em UTIN, observa a participação dos familiares no cuidado ao RN prematuro, considerando as sete medidas padrão neuroprotetoras?

### Análise e Tratamento dos Dados

Os dados provenientes dos formulários escritos e das entrevistas gravadas foram organizados em documentos digitais padronizados. As respostas textuais e os registros de áudio foram transcritos literal e integralmente, preservando o conteúdo original. Em seguida, as informações foram estruturadas em quadros e analisadas por meio da análise de conteúdo de Bardin^(9)^. Com a análise das entrevistas, observou-se que não houve perda de conteúdo, independentemente da forma de coleta do dado.

### Aspectos Eticos

O estudo foi aprovado pelo Comitê de Ética em Pesquisa da Universidade Federal de Alagoas, sob Parecer nº 7.182.417. Todos os profissionais assinaram o Termo de Consentimento Livre e Esclarecido, garantindo anonimato e voluntariedade. Para identificação dos participantes, obedecendo também ao critério do anonimato, optou-se por identificá-los com a letra “P”, seguida do número arábico, que foi realizada de forma crescente (P1, P2, P3, e assim sucessivamente).

## RESULTADOS

Participaram deste estudo 26 profissionais da equipe multiprofissional, em um universo de 300 profissionais, como médicos, enfermeiros, fisioterapeutas, fonoaudiólogos e técnicos de enfermagem. Os principais conteúdos variaram entre o impacto da internação na UTIN, a participação nos cuidados, os sentimentos dos pais, o vínculo e o acolhimento na UTIN, que, no Modelo de Cuidado Desenvolvimental, podem ser relacionados a uma das pétalas do lótus, na parceria com a família, e ao centro, com a díade mãe-bebê. Optou-se por apresentar e discutir os resultados a partir de duas categorias: “Entre o medo e a descoberta” e “Executando cuidados e promovendo o vínculo”.

### Entre o Medo e a Descoberta

O período de internação na UTIN é um desafio para os pais e bebês desde o seu início. Assim, o primeiro contato dos pais com o seu bebê ocorre, muitas vezes, na UTI, ambiente diferente e assustador, onde o profissional atua como mediador desse encontro:

*Acolhendo e oferecendo o contato inicial* (P4); *Porque eu acho que o ambiente da UTI, por ser UTI, é um nome que assusta, que deixa a pessoa com medo. Então, cada um tem seu tempo de criação de vínculo com a criança, porque ainda tem aquela questão do bebê ideal e do bebê real* (…) *ela chega na UTI e ainda precisa estabelecer vínculo, o que contribui aí com o desenvolvimento* (P21).

A partir de então, à medida que vão se inteirando do cotidiano e dos processos, aos poucos eles se adaptam ao ambiente, que ainda é novo, à rotina, que ainda é indefinida, e à situação clínica do RN, que é complexa. Por isso, o medo permeia sua estadia ao lado do bebê. Dos profissionais entrevistados, muitos citaram o “receio” que os pais apresentam, como pode ser visto nas falas:

*Tem medo do toque* (P3). *A família tem muito medo. Medo da perda, medo da piora, medo da gravidade, medo até em tocar o RN* (P24); *A família costuma observar* (P23); (…) *o primeiro impacto a gente sabe que é bem grande, né? Depois eles vão se ambientando, se acostumando* (P22).

Nesse contexto de superação do contato inicial e da condição materna, que ainda inspira atenção, a mãe se envolve no cuidado ao seu bebê e é incluída em uma nova unidade, onde se adapta aos horários de dieta e assistência ao seu bebê. Pouco a pouco, elas começam a se envolver se aproximar do RN:

*Elas têm receio, a gente pergunta se já sabe trocar a fraldinha, mas elas têm receio de mexer, porque a maioria deles estão monitorizados, aí elas ficam sem saber como vai manusear o bebê* (P17); (…) *mães com medo de terem tocado o bebê… a gente vai, conversa, estimula o toque, estimula este contato com o bebê* (…) *é um desafio* (P20).

Em todo o processo de internamento, independentemente do tempo de permanência na UTIN, destaca-se a importância da informação e dos benefícios da estadia dos pais junto ao bebê, tendo em vista que esse momento de internação também ocorre em um período de sensibilidade e fragilidade materna. Muitas vezes, esse é um marco para o estabelecimento de vínculo, e quanto mais informação estiverem disponíveis e mais por dentro eles estão das necessidades e cuidados que seu filho recebe, mais ameno se torna este momento:

*Informando sobre a doença e tirando dúvidas* (P6); *São pessoas extremamente ansiosas, carentes de informações e atenção. Ficam mais tranquilas e participam dos processos de cura sempre que são mais informados e acolhidos* (P7); *A maioria dos familiares se mantém presente, procura notícias e tem interesse em fazer a posição canguru* (P9); (…) *eu percebo mais na questão da amamentação, porque ela fica mais vinculada ao seu bebê, mas o pai é mais aquele de só olhar, observar* (P12).

Assim, conforme essa interação vai acontecendo, familiares e equipe se juntam em prol do cuidado ao RN, de forma a fortalecer a confiança, a participação e o engajamento na melhora do mesmo:

*Existe uma trajetória de aprendizado e mergulho junto com a equipe nos cuidados com o bebê — trajetória essa muito individual, em que a família está inserida nos cuidados e tem livre acesso aos seus filhos* (P8); *Participam em sua maioria de forma interativa dos cuidados com o RN, oferta de dieta, posição canguru, recebendo informações sobre o quadro clínico* (P10).

Um outro aspecto descrito pelos participantes foi em relação ao vínculo, tendo em vista a distância que a instabilidade do bebê, a quantidade de dispositivos, a própria incubadora e a necessidade de cuidados intensivos podem gerar. As mães ficam distantes dos cuidados que estariam ofertando aos seus bebês nesse período e, por isso, acabam tendo uma dificuldade de se visualizarem como mães naqueles momentos:

*Elas falam muito que, às vezes, elas acham que o filho não pertence a ela. Quando a gente coloca na posição canguru, muitas vezes já escutei: “É a primeira vez que eu estou sendo mãe do meu filho, que o meu filho pertence a mim”* (P11); (…) *eles ficam temerosos de ver o bebê dentro da incubadora. Mas é o mesmo bebê que eles teriam em casa com eles; o problema é que aqui precisa dos cuidados da gente, mas o bebê continua sendo deles* (P25).

A construção do vínculo, tão importante para o crescimento e o desenvolvimento infantil, acaba ocorrendo com relativo atraso, sendo galgada a passos menores, devido à necessidade de superação de receios, dúvidas e angústias:

*A posição canguru, né, tanto o pai quanto a mãe* (…) *a interação de tocar no bebê, conversar. Por se tratar de RN prematuro, algumas vezes eles ficam com medo, mas aí incentivo* (…) *porque é bom pros RNs, né? Pro desenvolvimento deles* (P18).

Uma das primeiras barreiras para a participação dos pais nos cuidados e na construção de vínculo com o RN na UTI é a condição clínica, tendo em vista sua instabilidade e a necessidade de cuidados intensivos, como pode ser visto na fala:

*No começo, elas ficam receosas. Porque ou eles estão no respirador ou a questão da intercorrência - muitas vezes tem intercorrência do bebê dessaturar, e aí elas acabam relacionando a instabilidade do bebê ao canguru, mas é realmente porque é o bebê mais grave* (P1).

Outros profissionais reforçam o que foi relatado anteriormente:

*E depois eles vão se adaptando àquele cuidado, e eles acabam perdendo o medo, se acostumam com aquele barulho, com aquela queda de saturação, quando eles começam a entender aquela situação* (P2).

Assim, pode-se observar, conforme a fala dos profissionais, que o choque inicial que o ambiente da UTIN exerce sobre os pais vai se tornando menos impactante conforme o período de internação. Os pais se mantêm em vigilância aos sintomas do bebê, mas a parceria com a equipe de saúde oportuniza que este momento seja contornado de forma mais amena, quando, aos poucos, eles são inseridos no cuidado e, ainda mais importante, informados durante o processo de internação.

### Executando Cuidados e Promovendo o Vínculo

Com suas rotinas diárias, as mães acabam ficando mais presentes ao lado do leito do bebê, devido ao processo de extração de leite para alimentá-lo e à possibilidade de internação hospitalar, considerando os benefícios da presença para o RN:

*Nossa maior aproximação é com as mães, que permanecem como acompanhantes no hospital e comparecem rotineiramente para a ordenha de leite humano e posicionamento canguru. E a equipe tem reconhecido cada vez mais a importância disso. Mas acho que é preciso avançar muito ainda no quesito participação dos cuidados* (P14); *A família é incluída no tratamento – imunoterapia e contato pele a pele* (P5).

A participação nos cuidados é relativa, variando de mãe para mãe. Mas vale ressaltar que, conforme a equipe reforça a importância da participação não apenas nos cuidados e na extração de leite, mas também na sua presença na unidade, já faz diferença para a saúde do bebê:

*Eu percebo que, se a gente instruir a importância deles participarem mais dos cuidados com o bebê, eles identificam a importância da presença deles e têm mais interesse em vir cuidar junto* (P19).

As oportunidades de participação nos cuidados com o bebê podem ser diversas, desde o toque e o posicionamento canguru até a troca de fraldas. Esses cuidados variam entre os específicos de uma unidade neonatal, como a oferta de dieta por sonda, e os próximos do cuidado familiar, como a troca de fraldas:

*Às vezes, acontece de, tipo assim, a gente estar oferecendo a dieta, e a gente pede pra eles segurarem a seringa naquele momento da dieta. Mas isso é raro na UTI. A participação deles é mesmo toque terapêutico e posição canguru* (P2).

Nesses momentos de participação, também surgem novas possibilidades, tendo em vista o papel que o profissional exerce em relação às orientações que pode oferecer à família em um cuidado mais peculiar:

*Eu peço ajuda a ela pra trocar uma fralda, aí eu ensino ela como é que troca uma fralda. Eu tento promover o máximo possível de colocar na posição canguru, de conversar com ela, dela entender que aquele filho é dela* (P11); *Precisam de orientações quanto às estratégias de favorecer a neuroproteção* (P26).

Além do pai e da mãe, que têm livre acesso à unidade e aos seus bebês, outros familiares têm acesso limitado às unidades para visita ao bebê, mas não têm muita participação nos cuidados do bebê:

*A família não tem acesso à unidade, apenas os avós ou algum responsável vinculado à família, uma vez por semana, o que impossibilita/dificulta a formação de vínculo/cuidado com a criança* (P15); (…) *são raros os casos que eu vejo as famílias se envolverem muito… são poucos casos* (P13).

A percepção dos profissionais em relação à participação no cuidado é diversa, variando conforme a disponibilidade dos pais, seus receios e a estabilidade clínica do bebê. Destaca-se na fala deles a participação principalmente na colocação do bebê na posição canguru e na extração do leite para oferta nas dietas. Os avós podem visitar os bebês, mas não participam de seus cuidados.

Para alguns profissionais, visualizar essa participação no cuidado ainda é difícil, tendo eles a leitura de que essa participação estaria mais tangível em outras unidades por onde o bebê passa, considerando a continuidade desse cuidado e a instabilidade do bebê:

*Na UTI, eu ainda acho muito pouco* (…) *eu acho que elas só começam a participar mais dos cuidados na canguru. Mas não é o correto, que a gente deveria promover esses cuidados desde a UTI* (P11).

Outro desafio se dá no contexto da orientação à família, como colocado:

*A maioria é a mãe que se envolve mais com tudo do bebê* (…) *e tem mães que são mais, às vezes até pela falta de informação, de orientação, que às vezes não tem. Falta, às vezes, essa informação* (…) *de direito, que às vezes elas têm e não são informadas sobre isso* (P14).

Mas isso se dá, muitas vezes, pelo grau de instrução daquela mulher/família:

*Recebemos mães/famílias de diferentes contextos… desde bebês que foram planejados até gravidez indesejada* (…) *com melhor e pior graus de instrução e renda* (P16).

Os profissionais apontam a necessidade de um olhar mais atencioso para as mães dos bebês, que se tornam mães acompanhantes de internação na unidade e têm toda uma rotina para a extração manual de leite à beira-leito a cada três horas, o que pode ser cansativo emocional e fisicamente. Alguns profissionais ainda enxergam uma participação mais restrita nos cuidados, enquanto outros relatam uma interação e troca maiores nos cuidados da mãe com seu filho. Por último, reforça-se sobre o papel da orientação para essa mãe/pai no contexto do cuidado, sendo importante o alinhamento da linguagem para a sua percepção.

## DISCUSSÃO

O nascimento prematuro do bebê faz com que a maternidade também ocorra prematuramente, não havendo tempo para a mulher assimilar e ajustar as representações do bebê que chegou, sendo necessário que ela se adapte a essa nova realidade. Após a separação não planejada, a mãe encontra à sua espera na UTIN um bebê frágil, magro e cercado por equipamentos, o que era diferente do que havia imaginado^(10)^.

A internação na UTIN é um desafio para os pais, pois compromete a dinâmica familiar e distancia os pais dos cuidados primários ao RN, gerando insegurança e impotência. Desenvolver a parentalidade cercada por ruídos, luzes constantes e pessoas estranhas, de fora de seu convívio, pode acabar transformando essa vivência^(11)^ e, por isso, ela pode ser permeada por diversos sentimentos, como frustração, culpa e sensação de distanciamento materno^(12)^. Isso corrobora o que foi dito pelos profissionais da pesquisa a respeito do medo da família sobre a internação em UTIN.

A necessidade de internação do bebê faz com que haja a mudança do papel de cuidador principal, sendo este assumido pelos profissionais de saúde, o que pode gerar nos pais os sentimentos de estresse, ansiedade e depressão^(13)^. Dessa forma, esse processo dificulta a formação do vínculo entre pais e bebês, como mencionado por P11, quando citou que as mães se sentem mais próximas quando colocam o bebê em posição canguru. A presença dos familiares na UTIN promove a interação entre mãe e bebê, o que traz impactos sobre o cérebro do recém-nascido pré-termo (RNPT). O contato pele a pele é tido como o melhor ambiente para o RN, pois cria um ambiente sensorial com interações benéficas para ambos, ajudando também a minimizar a dor e a promover o vínculo, além de facilitar a termorregulação^(8)^.

A ausência de informações sobre o estado clínico do bebê também pode distanciar as mães do seu filho, aumentando o nível de estresse e de ansiedade parental. Sendo assim, é primordial que os profissionais da unidade atuem como apoio, auxiliando-as a observar e a entender os sinais que o seu filho dá durante os momentos de interação de ambos^(14)^.

Com o passar do tempo entre a interação mãe-equipe e a rotina da unidade, é perceptível a mudança no comportamento materno, o que pode ser associado à superação dos sentimentos iniciais, ao acolhimento da equipe e à sensação de segurança, que ajuda a fortalecer o vínculo entre profissionais, familiares e crianças^(12)^. Entretanto, a instabilidade clínica do bebê, somada à possibilidade de ele não resistir ao internamento, faz com que haja ainda sentimentos de medo, insegurança e angústia. O sentimento pode ser ampliado a depender da condição de saúde da criança^(10)^.

Vale ressaltar que as políticas públicas garantem a participação dos pais nos cuidados com a criança no período de internação, desde o período de gestação, parto e pós-parto, com o pré-natal do parceiro e o direito ao acompanhante de livre escolha, bem como o livre acesso e permanência ao RN na unidade neonatal^(15)^. Não menos importante, as informações recebidas sobre o estado de saúde de seu bebê podem ajudá-los a compreender o processo de internação e, posteriormente, a assumir os cuidados em domicílio de forma mais segura^(14)^.

A participação dos pais nos cuidados é capaz de reduzir o impacto do internamento na UTIN para o desenvolvimento do RN, sendo um dos pilares do cuidado desenvolvimental. O contato pele a pele é uma medida que auxilia o alívio da dor, traz a sensação de segurança para o binômio, facilita a amamentação e reduz o estresse. Já a separação dos pais é capaz de afetar o desenvolvimento do RNPT e o estabelecimento do vínculo^(16)^.

A garantia da participação nos cuidados junto ao RNPT é um outro dado relevante para melhores desfechos. Em unidades com programas de cuidado parental, observou-se que houve redução do estresse familiar, aumento de taxas de aleitamento materno e melhores resultados de desenvolvimento neurológico e ganho de peso para o bebê. Com a participação ativa nos cuidados, foi evidenciada também aquisição de conhecimento sobre higiene e autoconfiança materna para o cuidado com o seu filho^(17)^.

Além disso, também pode ser citado o acesso às informações sobre o internamento do RN, que reduzem sentimentos negativos vivenciados pelos pais nesse período, fazendo com que se sintam participantes e preparados para o cuidado com o seu filho^(18)^. Autores que avaliaram o estresse parental de pais de bebês em UTIN evidenciaram que, quando cientes dos cuidados recebidos pelos seus filhos, houve um menor índice de estresse associado ao período de internação, incitando que, nesse período, os pais precisam de apoio, suporte e orientação por parte da equipe^(19)^.

Ademais, pesquisadores referiram também a importância de suporte às mães sobre como interagir com esse bebê, e não apenas sobre a condição clínica. Essas informações podem ajudar na redução da insegurança materna e no aumento de percepções positivas e do bem-estar. Outro fator benéfico é o método canguru, que fortalece a consciência do papel materno e estimula a comunicação precoce, contribuindo com as percepções favoráveis do período de internamento^(20)^.

As principais formas de participação das mães no cuidado ao bebê prematuro são a presença física, o acompanhamento dos cuidados exercidos pela equipe, a busca de informações sobre o bebê, os cuidados com a higiene e alimentação, o toque, segurar o bebê no colo e o posicionamento canguru^(21)^. Os profissionais entrevistados neste estudo afirmaram participar principalmente em relação ao posicionamento canguru, à oferta da dieta e à presença na unidade.

A inclusão dos pais no cuidado ao prematuro em UTIN ainda é um desafio, como descrito por muitos autores^(5,12,15)^. Entretanto, cabe à equipe multiprofissional da unidade atuar como intermediário para o estabelecimento de vínculo e de aproximação entre pais e bebê, contribuindo para a construção de um cuidado que fará com que a transição para a alta seja mais tranquila para a equipe e para a família^(22)^. Um dos eixos do cuidado desenvolvimental é a função da equipe de saúde de capacitar e promover essa participação, contribuindo para as práticas do modelo que tem o potencial de minimizar as consequências do internamento em UTIN^(17)^.

## CONCLUSÃO

A partir da perspectiva dos profissionais, a participação dos pais nos cuidados ao RN prematuro na UTIN é um processo complexo e dinâmico. Inicialmente, o medo e a incerteza dificultam o vínculo e a inserção nos cuidados. Essas barreiras são superadas pela atuação da equipe multiprofissional por meio de acolhimento, orientação contínua e incentivo a práticas como o método canguru, que transformam o medo em confiança.

No cerne do Modelo de Cuidado Desenvolvimental, apesar da relação direta com um dos sete eixos apresentados, a participação dos familiares é transversal a outras “pétalas” da “flor de lótus” que o representa, com o enriquecimento do ambiente sensorial e interacional do RNPT, a redução da dor e do estresse por meio do conforto, da amamentação e da redução da resposta à dor, a promoção da termorregulação, o contato pele a pele e o incentivo à amamentação.

Entretanto, persistem desafios, como a instabilidade clínica do RN, a participação limitada de familiares e influências socioeconômicas e institucionais. Conclui-se, assim, a necessidade de políticas de humanização que priorizem uma comunicação qualificada e apoio emocional, incluindo os pais como corresponsáveis desde a admissão. Essa abordagem é essencial para construir vínculos familiares estáveis e assegurar cuidados sustentáveis após a alta hospitalar.

## Data Availability

Todo o conjunto de dados que dá suporte aos resultados deste estudo faz parte de uma pesquisa de dissertação que ainda não foi publicizada em sua totalidade.

# Translation: Percepciones de los profesionales de las unidades neonatales sobre la participación de los padres en el cuidado de los bebés prematuros*

**DOI:** 10.1590/1980-220X-REEUSP-2025-0532es

**Authors:** Adrielly Cristina de Lima Raimundo, Ana Mirelle dos Santos, Paulyne Souza Silva Guimarães, Ingrid Martins Leite Lúcio, Ana Virgínia Rodrigues Veríssimo, Ana Carolina Santana Vieira, Rossana Teotônio de Farias Moreira

**Affiliations:** 1 Universidade Federal de Alagoas, Escola de Enfermagem, Programa de Pós-graduação em Enfermagem, Maceió, AL, Brasil.; 2 Universidade Federal de Alagoas, Escola de Enfermagem, Maceió, AL, Brasil.; 3 Universidade de Pernambuco, Faculdade de Enfermagem Nossa Senhora das Graças, Recife, PE, Brasil.

**Keywords:** Recien Nacido Prematuro, Unidades de Cuidado Intensivo Neonatal, Familia, Personal de Salud

## Abstract

**Objetivo:**

Describir la participación de los padres en el cuidado de recién nacidos prematuros desde la perspectiva de los profesionales de la Unidad de Cuidados Intensivos Neonatales.

**Método:**

Estudio descriptivo y cualitativo, realizado en marzo de 2025 con 26 profesionales del equipo multidisciplinario que trabajan en dos Unidades de Cuidados Intensivos Neonatales ubicadas en Maceió, Alagoas. El estudio fue presentado al Comité de Ética en Investigación de la Universidada Federal de Alagoas, bajo el Dictamen 7.182.417.

**Resultados:**

A partir de la lectura de las entrevistas, surgieron dos categorías: “Entre el miedo y el descubrimiento” y “Brindar cuidados y promover el vínculo”. Los profesionales informaron que la inclusión de los padres en la rutina de la unidad redujo el miedo y aumentó los beneficios para el bebé, especialmente con el método canguro.

**Conclusión:**

El miedo y la incertidumbre dificultan la participación de los padres en el cuidado. La acogida, la orientación y el fomento de estas prácticas por parte del equipo pueden ayudar a superar este obstáculo. Las políticas de humanización son necesarias para garantizar la corresponsabilidad en el cuidado.

## INTRODUCCIÓN

El nacimiento prematuro es un acontecimiento que plantea retos significativos para la familia y los profesionales de la salud, especialmente cuando el recién nacido (RN) necesita cuidados intensivos en Unidades de Cuidados Intensivos Neonatales (UCIN). Ante la complejidad de este escenario, crece la valoración de prácticas que promuevan la participación activa de los padres en el cuidado de su hijo prematuro, reconociéndolos como parte fundamental en el proceso de recuperación y desarrollo del niño^(1)^.

El proceso de hospitalización de un bebé en la UCIN es una experiencia estresante para los padres, lo que puede provocar emociones relacionadas con el miedo y la soledad ante la distancia y la falta de preparación. Sin embargo, cuando existe el apoyo de un equipo multiprofesional, este sentimiento puede ser sustituido por la autoconfianza^(2)^.

En este contexto, el papel del equipo es actuar como mediador entre el entorno técnico y la dimensión emocional de los padres. La forma en que los profesionales acogen, orientan e integran a la familia en el cuidado influye directamente en la calidad de la experiencia vivida por los padres durante la hospitalización de su hijo. Esta premisa constituye el núcleo de la Atención Centrada en la Familia, un modelo asistencial que reconoce a la familia como un socio esencial en el proceso de cuidado y recuperación del recién nacido^(3)^. Sin embargo, a pesar de los avances en las políticas de humanización, aún persisten barreras institucionales, culturales y comunicacionales que limitan la participación efectiva de los padres en el cuidado neonatal^(4)^.

Las prácticas de atención neonatal que trabajan con la inclusión y el acercamiento de la familia en el día a día del recién nacido durante la etapa de hospitalización en una unidad hospitalaria se han difundido con el objetivo de fortalecer el vínculo afectivo y el desarrollo infantil. En Brasil, el método canguro se considera una de las estrategias de atención humanizada para los recién nacidos en estado grave o potencialmente grave, y abarca planes de intervención biopsicosocial centrados en la atención al bebé y su familia^(5)^.

Sin embargo, el estudio también demuestra que la falta de preparación de las madres para cuidar al recién nacido que ha permanecido hospitalizado durante mucho tiempo en unidades neonatales puede afectar el cuidado del niño después del alta. Varios padres afirman sentir vacilación, temor e inexperiencia ante acciones de rutina relacionadas con su hijo, como el cambio de pañales. Esto demuestra que las orientaciones brindadas por los profesionales repercuten directamente en la salud del bebé en el entorno doméstico^(6)^.

En la rutina asistencial, se observa que el equipo multiprofesional de una UCIN, incluyendo al personal de enfermería, necesita fundamentos técnicos y científicos, así como capacitación, para potenciar la calidad de la atención a los recién nacidos y sus familiares. En el proceso de atención, es fundamental que el profesional comprenda la relación madre-bebé como algo positivo para la preservación y la rehabilitación de la salud del recién nacido, así como las necesidades de los padres, con el fin de brindar una atención individualizada^(7)^.

Así, el presente estudio busca responder a la siguiente pregunta: ¿cómo observa usted, como profesional que trabaja en una UCIN, la participación de los familiares en el cuidado del recién nacido prematuro, considerando las siete medidas neuroprotectoras estándar? Esto resulta relevante no solo para calificar las prácticas asistenciales, sino también para respaldar políticas públicas orientadas a la humanización de la atención y a la promoción de la salud integral del niño y su familia, con miras a contribuir a la construcción de un modelo de atención centrado en la familia y en la corresponsabilidad. Se propone, por lo tanto, describir la participación de los padres en el cuidado de los recién nacidos prematuros desde la perspectiva de los profesionales de la UCIN.

## MÉTODO

### Tipo de Estudio

Se trata de un estudio descriptivo, de perspectiva cualitativa, realizado con el equipo multiprofesional que trabaja en la UCIN a la luz del Modelo de Cuidado del Desarrollo de Altimier y Phillips^(8)^. Este modelo se aplicó en la elaboración del guion de entrevistas para la recolección de datos, en el análisis y en la discusión de los datos, centrándose en el eje de la participación de los padres. El modelo presenta una estructura que orienta la práctica clínica en la UCIN hacia la neuroprotección, a partir de siete medidas para el cuidado del desarrollo, representadas por una flor de loto. Estas medidas abarcan el entorno de curación, la optimización de la nutrición, la minimización del dolor y el estrés, la protección de la piel, la salvaguarda del sueño, el posicionamiento y la manipulación, y la colaboración con las familias, siendo la díada madre-bebé el centro de esta flor. El estudio siguió las recomendaciones de *COnsolidated criteria for REporting Qualitative research (Criterios consolidados para la presentación de informes de investigación cualitativa)*.

### Lugar, Población y Criterios de Selección

Este estudio se llevó a cabo en dos UCIN de dos hospitales universitarios, que, en conjunto, suman 36 camas, atendidas por el Sistema Único de Salud, ambos ubicados en Maceió, Alagoas, Brasil. Se incluyeron en el estudio los profesionales del equipo multiprofesional que prestan asistencia directa en el cuidado del recién nacido, como médicos, enfermeros, fisioterapeutas, fonoaudiólogos y técnicos de enfermería, y que llevaran al menos un año en el cargo.

### Recolección de Datos

La recolección de datos se llevó a cabo en marzo de 2025, mediante entrevistas individuales con profesionales de la salud.

Se optó por un enfoque de muestreo por conveniencia, respetando la disponibilidad de los participantes durante el período de Recolección: la investigadora acudía a las unidades dos veces por semana y, de acuerdo con el turno de guardia vigente, presentaba la propuesta del estudio e invitaba a los profesionales a participar voluntariamente. Todas las entrevistas se realizaron de manera individual, en un lugar reservado, garantizando la confidencialidad y el anonimato de los participantes.

Teniendo en cuenta la rutina de las unidades y la necesidad de reducir posibles incomodidades, las entrevistas se llevaron a cabo de dos formas: por escrito, entregando el formulario para que el profesional lo devolviera posteriormente completado; o grabadas, con captura de audio durante la aplicación del guion, previamente presentado, informado y autorizado por el profesional. Ambas formas de entrevista siguieron el mismo guion de preguntas. Se utilizó como pregunta inicial: ¿cómo observa usted, como profesional que trabaja en la UCIN, la participación de los familiares en el cuidado del recién nacido prematuro, considerando las siete medidas neuroprotectoras estándar?

### Análisis y Tratamiento de los Datos

Los datos procedentes de los formularios escritos y de las entrevistas grabadas se organizaron en documentos digitales estandarizados. Las respuestas textuales y los registros de audio se transcribieron literal e íntegramente, preservando el contenido original. A continuación, la información se estructuró en cuadros y se analizó mediante el análisis de contenido de Bardin^(9)^. Con el análisis de las entrevistas, se observó que no hubo pérdida de contenido, independientemente de la forma de recolección de los datos.

### Aspectos Eticos

El estudio fue aprobado por el Comité de Ética en Investigación de la Universidad Federal de Alagoas, mediante el Dictamen n.º 7.182.417. Todos los profesionales firmaron el Término de Consentimiento Libre e Informado, garantizando el anonimato y la voluntariedad. Para la identificación de los participantes, respetando también el criterio de anonimato, se optó por identificarlos con la letra “P”, seguida del número arábigo, que se asignó de forma ascendente (P1, P2, P3, y así sucesivamente).

## RESULTADOS

Participaron en este estudio 26 profesionales del equipo multiprofesional, de un total de 300 profesionales, entre los que se encontraban médicos, enfermeros, fisioterapeutas, fonoaudiólogos y técnicos de enfermería. Los principales contenidos variaron entre el impacto de la hospitalización en la UCIN, la participación en los cuidados, los sentimientos de los padres, el vínculo y la acogida en la UCIN, que, en el Modelo de Cuidado del Desarrollo, pueden relacionarse con uno de los pétalos del loto, en la colaboración con la familia, y con el centro, con la díada madre-bebé. Se optó por presentar y discutir los resultados a partir de dos categorías: “Entre el miedo y el descubrimiento” y “Brindando cuidados y promoviendo el vínculo”.

### Entre el Miedo y el Descubrimiento

El período de hospitalización en la UCIN es un desafío para los padres y los bebés desde el principio. Así, el primer contacto de los padres con su bebé ocurre, muchas veces, en la UCI, un ambiente diferente y aterrador, donde el profesional actúa como mediador de ese encuentro:

*Acogiendo y ofreciendo el contacto inicial* (P4); *Porque creo que el ambiente de la UCI, por ser UCI, es un nombre que asusta, que deja a la persona con miedo*.

*Entonces, cada uno tiene su tiempo para crear un vínculo con el niño, porque todavía está esa cuestión del bebé ideal y del bebé real* (…) *ella llega a la UCI y aún necesita establecer un vínculo, lo que contribuye al desarrollo* (P21).

A partir de ahí, a medida que se van familiarizando con el día a día y los procesos, poco a poco se adaptan al entorno, que aún es nuevo, a la rutina, que aún es indefinida, y a la situación clínica del recién nacido, que es compleja. Por eso, el miedo impregna su estancia junto al bebé. De los profesionales entrevistados, muchos mencionaron el ‘‘temor” que presentan los padres, como se puede ver en los siguientes comentarios:

*Tienen miedo al contacto físico* (P3). *La familia tiene mucho miedo. Miedo a la pérdida, miedo a que empeore, miedo a la gravedad, miedo incluso a tocar al recién nacido* (P24); *La familia suele observar* (P23); (…) *sabemos que el primer impacto es muy grande, ¿no? Después se van ambientando, acostumbrándose* (P22).

En este contexto de superación del contacto inicial y de la condición materna, que aún requiere atención, la madre se involucra en el cuidado de su bebé y es incluida en una nueva unidad, donde se adapta a los horarios de alimentación y atención de su bebé. Poco a poco, comienzan a involucrarse y a acercarse al recién nacido:

*Tienen miedo, les preguntamos si ya saben cambiar el pañal, pero tienen miedo de tocarlo, porque la mayoría están conectados a monitores, y entonces no saben cómo manejar al bebé* (P17); (…) *madres con miedo de haber tocado al bebé… nosotras vamos, hablamos, estimulamos el contacto físico, estimulamos este contacto con el bebé* (…) *es un desafío* (P20).

A lo largo de todo el proceso de hospitalización, independientemente del tiempo de permanencia en la UCIN, se destaca la importancia de la información y los beneficios de la estancia de los padres junto al bebé, teniendo en cuenta que este momento de hospitalización también ocurre en un período de sensibilidad y fragilidad materna. Muchas veces, este es un hito para el establecimiento del vínculo, y cuanta más información haya disponible y más al tanto estén de las necesidades y cuidados que recibe su hijo, más llevadero se vuelve este momento:

*Informando sobre la enfermedad y aclarando dudas* (P6); *Son personas extremadamente ansiosas, necesitadas de información y atención. Se tranquilizan y participan en los procesos de curación siempre que están más informadas y son acogidas* (P7); *La mayoría de los familiares se mantiene presente, busca noticias y tiene interés en practicar la posición canguro* (P9); (…) *lo noto más en el tema de la lactancia, porque ella se vincula más con su bebé, pero el padre es más de solo mirar, observar* (P12).

Así, a medida que se produce esta interacción, los familiares y el equipo se unen en pro del cuidado del recién nacido, con el fin de fortalecer la confianza, la participación y el compromiso con su mejoría:

*Existe un proceso de aprendizaje y de inmersión junto con el equipo en el cuidado del bebé —un proceso muy individual, en el que la familia se integra en los cuidados y tiene libre acceso a sus hijos* (P8); *La mayoría participa de manera interactiva en el cuidado del recién nacido, la alimentación, la posición canguro y la recepción de información sobre el cuadro clínico* (P10).

Otro aspecto descrito por los participantes fue el vínculo, teniendo en cuenta la distancia que pueden generar la inestabilidad del bebé, la cantidad de dispositivos, la propia incubadora y la necesidad de cuidados intensivos. Las madres se mantienen alejadas de los cuidados que estarían brindando a sus bebés en este período y, por eso, terminan teniendo dificultades para verse a sí mismas como madres en esos momentos:

*Hablan mucho de que, a veces, sienten que el hijo no les pertenece. Cuando lo colocamos en la posición canguro, muchas veces he escuchado: “Es la primera vez que soy madre de mi hijo, que mi hijo me pertenece”* (P11); (…) *les da miedo ver al bebé dentro de la incubadora. Pero es el mismo bebé que tendrían en casa con ellas; el problema es que aquí necesita nuestros cuidados, pero el bebé sigue siendo suyo* (P25).

La construcción del vínculo, tan importante para el crecimiento y el desarrollo infantil, termina ocurriendo con un retraso relativo, y se va logrando a pasos más pequeños, debido a la necesidad de superar temores, dudas y angustias:

*La posición canguro, ¿no?, tanto el papá como la mamá* (…) *la interacción de tocar al bebé, hablarle. Al tratarse de recién nacidos prematuros, a veces tienen miedo, pero entonces los animo* (…) *porque es bueno para los recién nacidos, ¿no? Para su desarrollo* (P18).

Una de las primeras barreras para la participación de los padres en los cuidados y en la construcción del vínculo con el recién nacido en la UCI es la condición clínica, dada su inestabilidad y la necesidad de cuidados intensivos, como se puede ver en el siguiente comentario:

*Al principio, se muestran temerosos. Porque o bien están con respirador o bien por la cuestión de las complicaciones —a menudo hay complicaciones en las que el bebé se desatura—, y entonces terminan relacionando la inestabilidad del bebé con el método canguro, pero en realidad es porque el bebé está más grave* (P1).

Otros profesionales refuerzan lo relatado anteriormente:

*Luego se van adaptando a ese cuidado, y terminan perdiendo el miedo, se acostumbran a ese ruido, a esa caída de la saturación, cuando empiezan a entender esa situación* (P2).

Así, se puede observar, según el discurso de los profesionales, que el impacto inicial que el entorno de la UCIN ejerce sobre los padres se va atenuando a medida que avanza el período de hospitalización.

Los padres se mantienen atentos a los síntomas del bebé, pero la colaboración con el equipo de salud permite que este momento se supere de manera más llevadera, cuando, poco a poco, se les incluye en los cuidados y, lo que es más importante, se les informa durante el proceso de hospitalización.

### Brindando Cuidados y Promoviendo el Vínculo

Con sus rutinas diarias, las madres terminan pasando más tiempo junto a la cuna del bebé, debido al proceso de extracción de leche para alimentarlo y a la posibilidad de hospitalización, considerando los beneficios de su presencia para el recién nacido:

*Nuestro mayor acercamiento es con las madres, que permanecen como acompañantes en el hospital y acuden de manera rutinaria para la extracción de leche materna y el contacto piel con piel. Y el equipo ha reconocido cada vez más la importancia de esto. Pero creo que aún hay que avanzar mucho en cuanto a la participación en los cuidados* (P14); *Se incluye a la familia en el tratamiento: inmunoterapia y contacto piel con piel* (P5).

La participación en los cuidados es relativa y varía de una madre a otra. Pero vale la pena destacar que, a medida que el equipo refuerza la importancia de la participación no solo en los cuidados y en la extracción de leche, sino también en su presencia en la unidad, esto ya marca una diferencia para la salud del bebé:

*Me doy cuenta de que, si les enseñamos la importancia de que participen más en los cuidados del bebé, ellos identifican la importancia de su presencia y tienen más interés en venir a cuidar juntos* (P19).

Las oportunidades de participación en el cuidado del bebé pueden ser diversas, desde el contacto físico y el método canguro hasta el cambio de pañales. Estos cuidados varían entre los específicos de una unidad neonatal, como la alimentación por sonda, y los más cercanos al cuidado familiar, como el cambio de pañales:

*A veces sucede que, por ejemplo, estamos administrando la alimentación y les pedimos que sostengan la jeringa en ese momento. Pero eso es raro en la UCI. Su participación es realmente el contacto terapéutico y la posición canguro* (P2).

En esos momentos de participación, también surgen nuevas posibilidades, teniendo en cuenta el papel que el profesional desempeña en relación con las orientaciones que puede ofrecer a la familia en un cuidado más particular:

*Le pido ayuda para cambiarle un pañal, y luego le enseño cómo se cambia un pañal. Intento promover al máximo que lo coloquen en la posición canguro, que hablen con él, que entiendan que ese hijo es suyo* (P11); *Necesitan orientación sobre estrategias para favorecer la neuroprotección* (P26).

Además del padre y la madre, que tienen libre acceso a la unidad y a sus bebés, otros familiares tienen acceso limitado a las unidades para visitar al bebé, pero no participan mucho en su cuidado:

*La familia no tiene acceso a la unidad, solo los abuelos o algún responsable vinculado a la familia, una vez por semana, lo que imposibilita o dificulta la formación de un vínculo o el cuidado con el niño* (P15); (…) *son raros los casos en los que veo a las familias involucrarse mucho… son pocos casos* (P13).

La percepción de los profesionales respecto a la participación en el cuidado es diversa, y varía según la disponibilidad de los padres, sus temores y la estabilidad clínica del bebé. En sus comentarios se destaca principalmente la participación en colocar al bebé en la posición canguro y en la extracción de leche para ofrecerla en las dietas. Los abuelos pueden visitar a los bebés, pero no participan en su cuidado.

Para algunos profesionales, visualizar esta participación en el cuidado sigue siendo difícil, ya que consideran que esta participación sería más tangible en otras unidades por las que pasa el bebé, teniendo en cuenta la continuidad de este cuidado y la inestabilidad del bebé:

*En la UCI, todavía me parece muy poco* (…) *creo que ellas solo empiezan a participar más en los cuidados en la posición canguro. Pero no es lo correcto, deberíamos promover estos cuidados desde la UCI* (P11).

Otro desafío se da en el contexto de la orientación a la familia, como se señala:

*La mayoría de las veces es la madre la que se involucra más en todo lo relacionado con el bebé* (…) *y hay madres que son más, a veces incluso por falta de información, de orientación, que a veces no tienen. A veces falta esa información* (…) *sobre sus derechos, que a veces tienen y no se les informa al respecto* (P14).

Pero esto se debe, a menudo, al nivel de educación de esa mujer/familia:

*Recibimos a madres/familias de diferentes contextos… desde bebés que fueron planeados hasta embarazos no deseados* (…) *con mejores y peores niveles de educación e ingresos* (P16).

Los profesionales señalan la necesidad de una mirada más atenta hacia las madres de los bebés, que se convierten en madres acompañantes durante la hospitalización en la unidad y tienen toda una rutina de extracción manual de leche junto a la cama cada tres horas, lo que puede ser agotador tanto emocional como físicamente. Algunos profesionales aún perciben una participación más limitada en los cuidados, mientras que otros informan de una mayor interacción e intercambio en los cuidados de la madre con su hijo. Por último, se refuerza el papel de la orientación para esta madre/padre en el contexto del cuidado, siendo importante la adaptación del lenguaje a su percepción.

## DISCUSIÓN

El nacimiento prematuro del bebé hace que la maternidad también se produzca prematuramente, sin que la mujer tenga tiempo para asimilar y ajustar las representaciones del bebé que ha llegado, siendo necesario que se adapte a esta nueva realidad. Tras la separación no planeada, la madre se encuentra en la UCIN con un bebé frágil, delgado y rodeado de equipos, lo cual era diferente de lo que había imaginado^(10)^.

La hospitalización en la UCIN es un desafío para los padres, ya que compromete la dinámica familiar y aleja a los padres de los cuidados primarios del recién nacido, generando inseguridad e impotencia.

Desarrollar la parentalidad rodeada de ruidos, luces constantes y personas extrañas, ajenas a su círculo, puede terminar transformando esta experiencia^(11)^ y, por ello, puede estar impregnada de diversos sentimientos, como frustración, culpa y sensación de distanciamiento materno^(12)^. Esto corrobora lo que dijeron los profesionales de la investigación con respecto al miedo de la familia ante la hospitalización en la UCIN.

La necesidad de hospitalizar al bebé provoca un cambio en el papel de cuidador principal, que pasa a ser asumido por los profesionales de la salud, lo que puede generar en los padres sentimientos de estrés, ansiedad y depresión^(13)^. De este modo, este proceso dificulta la formación del vínculo entre padres y bebés, como mencionó P11, al señalar que las madres se sienten más cercanas cuando colocan al bebé en posición canguro. La presencia de los familiares en la UCIN promueve la interacción entre la madre y el bebé, lo que tiene un impacto en el cerebro del recién nacido prematuro (RNPT). El contacto piel con piel se considera el mejor entorno para el recién nacido, ya que crea un ambiente sensorial con interacciones beneficiosas para ambos, lo que también ayuda a minimizar el dolor y a promover el vínculo, además de facilitar la termorregulación^(8)^.

La falta de información sobre el estado clínico del bebé también puede distanciar a las madres de su hijo, aumentando el nivel de estrés y ansiedad parental. Por lo tanto, es fundamental que los profesionales de la unidad actúen como apoyo, ayudándolas a observar y comprender las señales que su hijo da durante los momentos de interacción entre ambos^(14)^.

Con el paso del tiempo, entre la interacción entre la madre y el equipo y la rutina de la unidad, se percibe un cambio en el comportamiento materno, lo que puede asociarse a la superación de los sentimientos iniciales, a la acogida del equipo y a la sensación de seguridad, que ayuda a fortalecer el vínculo entre profesionales, familiares y niños^(12)^. Sin embargo, la inestabilidad clínica del bebé, sumada a la posibilidad de que no resista la hospitalización, hace que aún persistan sentimientos de miedo, inseguridad y angustia. Este sentimiento puede intensificarse dependiendo del estado de salud del niño^(10)^.

Cabe destacar que las políticas públicas garantizan la participación de los padres en el cuidado del niño durante el período de hospitalización, desde la gestación, el parto y el posparto, con la atención prenatal de la pareja y el derecho a un acompañante de libre elección, así como el libre acceso y permanencia junto al recién nacido en la unidad neonatal^(15)^. No menos importante, la información recibida sobre el estado de salud de su bebé puede ayudarlos a comprender el proceso de hospitalización y, posteriormente, a asumir los cuidados en el hogar de manera más segura^(14)^.

La participación de los padres en los cuidados puede reducir el impacto de la hospitalización en la UCIN sobre el desarrollo del recién nacido, siendo uno de los pilares del cuidado del desarrollo. El contacto piel con piel es una medida que ayuda a aliviar el dolor, aporta una sensación de seguridad a la pareja, facilita la lactancia materna y reduce el estrés. Por el contrario, la separación de los padres puede afectar el desarrollo del recién nacido prematuro y el establecimiento del vínculo^(16)^.

Garantizar la participación en los cuidados del recién nacido prematuro es otro factor relevante para obtener mejores resultados. En unidades con programas de cuidado parental, se observó una reducción del estrés familiar, un aumento de las tasas de lactancia materna y mejores resultados en el desarrollo neurológico y el aumento de peso del bebé. Con la participación activa en los cuidados, también se evidenció la adquisición de conocimientos sobre higiene y la autoconfianza materna para el cuidado de su hijo^(17)^.

Además, también cabe mencionar el acceso a la información sobre la hospitalización del recién nacido, lo que reduce los sentimientos negativos que experimentan los padres durante este período, haciéndolos sentir participantes y preparados para el cuidado de su hijo^(18)^. Los autores que evaluaron el estrés parental de los padres de bebés en la UCIN pusieron de manifiesto que, cuando eran conscientes de los cuidados que recibían sus hijos, se observaba un menor índice de estrés asociado al período de hospitalización, lo que sugiere que, en ese período, los padres necesitan apoyo, respaldo y orientación por parte del equipo^(19)^.

Además, los investigadores también señalaron la importancia de brindar apoyo a las madres sobre cómo interactuar con el bebé, y no solo sobre la condición clínica. Esta información puede ayudar a reducir la inseguridad materna y a aumentar las percepciones positivas y el bienestar. Otro factor beneficioso es el método canguro, que fortalece la conciencia del papel materno y estimula la comunicación temprana, contribuyendo a percepciones favorables del período de hospitalización^(20)^.

Las principales formas de participación de las madres en el cuidado del bebé prematuro son la presencia física, el seguimiento de los cuidados prestados por el equipo, la búsqueda de información sobre el bebé, los cuidados de higiene y alimentación, el contacto físico, sostener al bebé en el regazo y la posición canguro^(21)^. Los profesionales entrevistados en este estudio afirmaron participar principalmente en relación con la posición canguro, la alimentación y la presencia en la unidad.

La inclusión de los padres en el cuidado del bebé prematuro en la UCIN sigue siendo un desafío, tal como lo describen muchos autores^(5,12,15)^. Sin embargo, corresponde al equipo multiprofesional de la unidad actuar como intermediario para establecer el vínculo y el acercamiento entre los padres y el bebé, contribuyendo a la construcción de un cuidado que hará que la transición al alta sea más tranquila tanto para el equipo como para la familia^(22)^. Uno de los ejes del cuidado del desarrollo es la función del equipo de salud de capacitar y promover esta participación, contribuyendo a las prácticas del modelo que tiene el potencial de minimizar las consecuencias de la hospitalización en la UCIN^(17)^.

## CONCLUSIÓN

Desde la perspectiva de los profesionales, la participación de los padres en el cuidado del recién nacido prematuro en la UCIN es un proceso complejo y dinámico. Inicialmente, el miedo y la incertidumbre dificultan el vínculo y la incorporación a los cuidados. Estas barreras se superan gracias a la actuación del equipo multiprofesional mediante la acogida, la orientación continua y el fomento de prácticas como el método canguro, que transforman el miedo en confianza.

En el núcleo del Modelo de Cuidado del Desarrollo, a pesar de la relación directa con uno de los siete ejes presentados, la participación de los familiares es transversal a otros “pétalos” de la “flor de loto” que lo representa, con el enriquecimiento del entorno sensorial e interactivo del recién nacido prematuro, la reducción del dolor y el estrés mediante el confort, la lactancia materna y la reducción de la respuesta al dolor, la promoción de la termorregulación, el contacto piel con piel y el fomento de la lactancia materna.

Sin embargo, persisten desafíos, como la inestabilidad clínica del recién nacido, la participación limitada de los familiares y las influencias socioeconómicas e institucionales. Se concluye, por lo tanto, la necesidad de políticas de humanización que prioricen una comunicación calificada y el apoyo emocional, incluyendo a los padres como corresponsables desde el ingreso. Este enfoque es esencial para construir vínculos familiares estables y asegurar cuidados sostenibles tras el alta hospitalaria.

## Data Availability

Todo el conjunto de datos que respalda los resultados de este estudio forma parte de una investigación de tesis que aún no se ha publicado en su totalidad.
